# Ultrasonographic measurement of renal length and cortical thickness in clinically healthy Korean raccoon dogs

**DOI:** 10.3389/fvets.2025.1616776

**Published:** 2025-08-08

**Authors:** Moonsun Jang, Myeongsu Kim, Jae-Ik Han, Kichang Lee, Hakyoung Yoon

**Affiliations:** ^1^Department of Veterinary Medical Imaging, College of Veterinary Medicine, Jeonbuk National University, Iksan, Republic of Korea; ^2^Laboratory of Exotic and Wild Animal Medicine, College of Veterinary Medicine, Jeonbuk National University, Iksan, Republic of Korea

**Keywords:** ultrasonography, renal length, cortical thickness, aortic diameter, Korean raccoon dog, wildlife imaging

## Abstract

**Introduction:**

Ultrasonographic assessment of renal parameters is a useful noninvasive tool for evaluating kidney health. Although reference values for renal size have been established in domestic species such as dogs and cats, corresponding data are unavailable for wild Korean raccoon dogs (*Nyctereutes procyonoides koreensis*), an endemic canid species in Korea.

**Methods:**

This retrospective study analyzed abdominal ultrasonographic images from 36 clinically healthy wild Korean raccoon dogs. Measurements included renal length, renal cortical thickness (RCT), and aortic diameter, obtained using standardized imaging protocols. The ratios of renal length-to-aortic diameter (length/Ao) and RCT-to-aortic diameter (RCT/Ao) were calculated. Statistical analyses included paired and independent *t*-tests and Pearson correlation coefficients to assess differences and relationships among variables.

**Results:**

The mean renal length was 49.17 ± 3.53 mm for the left kidney, 47.53 ± 3.83 mm for the right kidney, and 48.36 ± 3.75 mm when both kidneys were pooled. The mean length/Ao ratios of the left and right kidneys were 10.53 ± 1.49 and 10.13 ± 1.53, respectively. The mean RCT for both kidneys was 4.81 ± 0.58 mm, and the mean RCT/Ao ratio was 1.03 ± 0.18. No significant sex-based differences were observed. Aortic diameter showed a positive correlation with body weight, whereas both length/Ao and RCT/Ao ratios were negatively correlated with body weight.

**Conclusion:**

This study provides the first ultrasonographic reference values for renal length, cortical thickness, and their corresponding aortic diameter ratios in wild Korean raccoon dogs. These findings offer foundational data for renal imaging assessment in this species and contribute to the development of species-specific reference standards in wildlife veterinary medicine.

## 1 Introduction

The kidneys are a pair of retroperitoneal organs crucial for excretion, metabolism, secretion, and regulatory functions. Acute kidney injury (AKI) and chronic kidney disease (CKD) lead to structural changes and functional deterioration of the kidneys. AKI may cause transient kidney enlargement due to cortical swelling, whereas CKD is associated with progressive renal atrophy, fibrosis, and a decrease in renal cortical thickness (RCT) and renal length (1–4). In dogs, renal size can change depending on the progression of renal disease (5). In particular, human studies have shown that the renal cortex responds more sensitively to pathological changes than the medulla, with cortical thinning appearing in the early stages of renal dysfunction (6). Accordingly, recent veterinary research in dogs and cats has increasingly focused on evaluating the renal cortex using parameters such as the RCT and RCT-to-aortic diameter (RCT/Ao) ratio (2, 7).

The Korean raccoon dog (*Nyctereutes procyonoides koreensis*), a subspecies of the Canidae family, is endemic to the Korean Peninsula in East Asia and is widely distributed across South Korea (8). The population of wild Korean raccoon dogs has rapidly increased due to factors such as the decline in natural predators (9). Although the number of studies on the veterinary radiology of Korean raccoon dogs has increased (10, 11), ultrasonographic research on their abdominal organs remains limited.

Abdominal ultrasonography is a valuable imaging tool for evaluating the renal size and has several advantages over other radiographic methods. Unlike radiography using ionizing radiation, ultrasonography is free from known biological hazards, does not require intravenous contrast administration, and does not depend on renal function. Furthermore, ultrasonography enables the quantitative measurements of RCT, renal length, and parenchymal structure, making it an essential tool for assessing renal disease progression (12). Renal size measurements are expected to be useful for detecting renal diseases in wild Korean raccoon dogs. Although normal reference ranges for renal size and RCT have been established for domestic dogs (*Canis lupus familiaris*) (7), no such data are yet available for Korean raccoon dogs, also a member of the Canidae family. Despite the availability of renal size data in dogs, ultrasonographic studies in Korean raccoon dogs are limited, and no species-specific reference values have been established.

We aimed to establish baseline ultrasonographic reference values for renal length, RCT, and length-to-aortic diameter (length/Ao) and RCT/Ao ratios in clinically healthy wild Korean raccoon dogs.

## 2 Materials and methods

### 2.1 Experimental design and subject selection criteria

The clinical records of wild Korean raccoon dogs rescued at the Jeonbuk Wildlife Rescue Center and treated at the Jeonbuk National University Animal Medical Center between November 2021 and January 2025 were retrospectively collected. Only Korean raccoon dogs with complete medical records—including physical examination (consisting of visual inspection of general appearance, auscultation, body temperature measurement, respiratory rate assessment, and evaluation of mental status), hematological and serum biochemical analysis (including blood urea nitrogen and creatinine), and abdominal ultrasonography within 1 week—and no abnormal findings on these assessments, were included as clinically healthy individuals.

All ultrasonographic examinations were performed without sedation, as manual restraint was sufficient for image acquisition in all cases.

The final study population comprised 36 adult (weaned) Korean raccoon dogs. Among the 36 Korean raccoon dogs included in this study, the primary reasons for rescue were external parasitic infection (77.8%), trauma (19.4%), and being found as strays (2.8%).

This study was approved by the Institutional Animal Care and Use Committee of Jeonbuk National University, Iksan-si, Jeollabuk-do, Republic of Korea (Approval no. NON2023-156).

### 2.2 Ultrasound measurements and analysis

**Figure 1:**
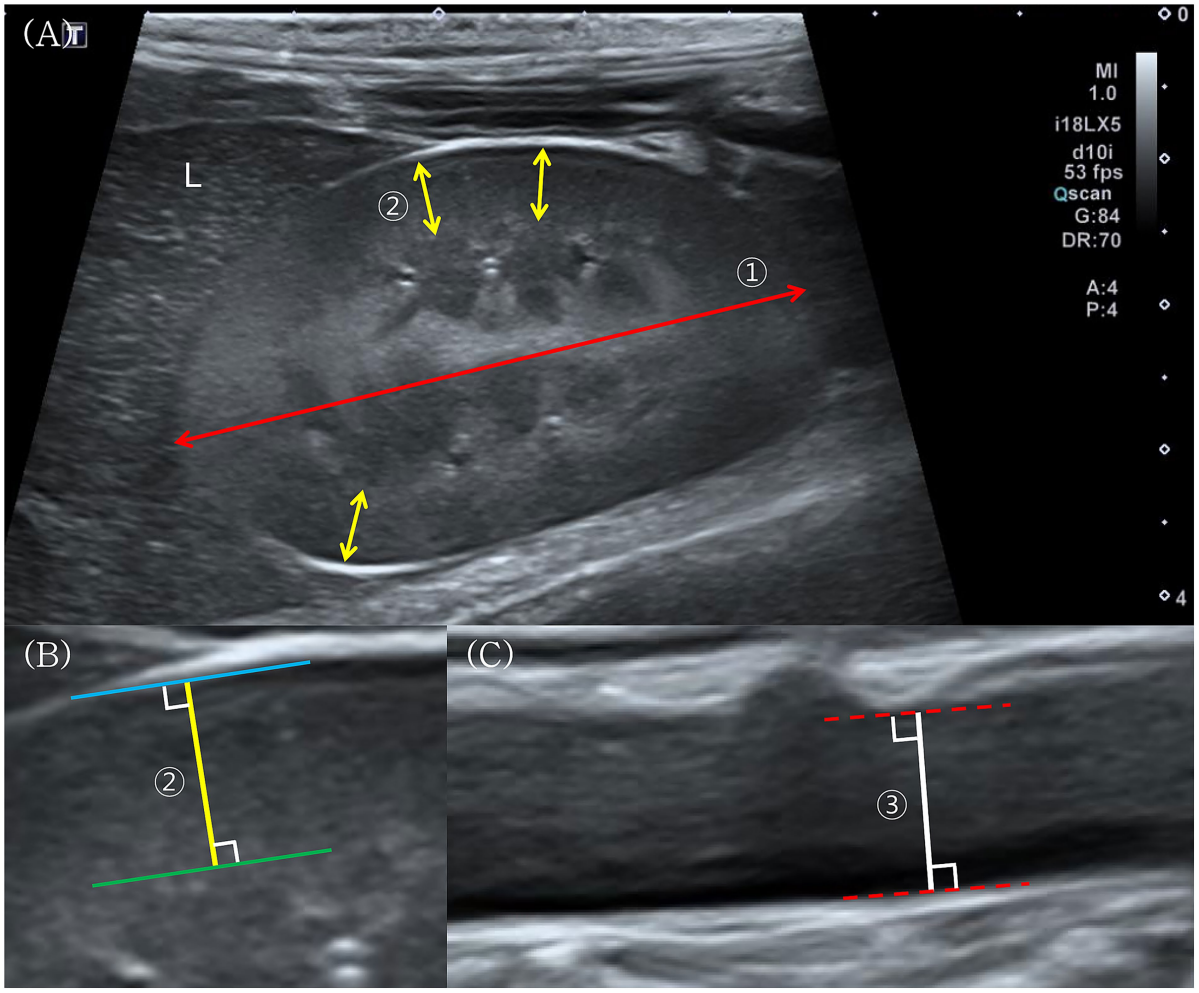
Ultrasonographic measurement of renal length, renal cortical thickness (RCT), and aortic diameter in wild Korean raccoon dogs. **(A)** Longitudinal ultrasonographic image of the right kidney, which is visualized adjacent to the caudate process of the liver (L). Renal length ① is defined as the longest linear distance between the cranial and caudal poles (red double-headed arrow). RCT ② is measured at sites where the base of the medullary pyramid is clearly visible (yellow double-headed arrows). **(B)** Magnified image of an RCT measurement. RCT is further measured as the shortest distance between the leading edge of the base of the medullary pyramid (green solid line) and the trailing edge of the renal capsule (blue solid line). **(C)** Longitudinal ultrasonographic image of the abdominal Aorta. The aortic diameter ③ measured at a level caudal to the left renal artery branching, is defined as the maximal intraluminal diameter between the inner edges of the aortic lumens (red dotted lines).

Ultrasonographic examinations were performed using an Aplio 300 (Canon Medical Systems, Zoetermeer, Netherlands) or Aplio i800 (Canon Medical Systems, Tokyo, Japan) equipped with a 12 MHz linear probe (18L7 or i18LX5). Each animal was placed in dorsal recumbency, and a ventrolateral approach was applied to obtain sagittal images of both kidneys and the Aorta (Figure 1).

RCT was measured following previously reported methods in dogs (2, 7). The shortest distance from the base of the medullary pyramid to the renal capsule was measured three times on the sagittal plane and averaged for each kidney. Aortic diameter was measured in the sagittal plane at the level caudal to the left renal artery branching, capturing the maximal intraluminal diameter between the lumen edges. Renal length was measured by identifying the longest linear distance between the cranial and caudal poles on the midsagittal plane of each kidney.

All ultrasound images were acquired by three observers (including MJ and two trained assistants), whereas all measurements and analyses were performed by a single radiology resident (MJ), who is in the third year of training. Final decisions regarding inclusion or exclusion were made by consensus between two senior veterinary radiologists (KL, Ph. D.; HY, Ph. D.), each with more than 10 years of experience.

### 2.3 Data analysis

Statistical methods were partially adopted from earlier canine studies (2, 7). Statistical analyses were performed using SPSS Statistics for Windows (version 20.0; IBM Corp., Armonk, NY, Unites States). Statistical significance was set at *p* < 0.05. The normality of data distribution was assessed using the Shapiro–Wilk test. Since all variables followed a normal distribution, the data were expressed as mean ± standard deviation.

A paired *t*-test was conducted to compare kidney length and RCT between the left and right kidneys, whereas an independent *t*-test assessed differences between sexes.

Correlations between body weight (BW) and renal parameters (kidney length and RCT) were evaluated using Pearson’s correlation analysis. Since no significant correlation was observed, no further regression analysis was performed.

In addition, Pearson’s correlation analysis was performed to assess the relationship between BW and aortic diameter, length/Ao ratio, and RCT/Ao ratio.

## 3 Results

Abdominal ultrasonography was performed on 36 clinically healthy wild Korean raccoon dogs. The study population comprised 21 males and 15 females, with a total of 72 renal ultrasound images analyzed. BWs ranged from 2.55 to 5.90 kg, with a mean of 4.02 ± 0.73 kg. Renal length was measured in both kidneys of all 36 raccoon dogs. Additionally, RCT and aortic diameter were measured in 30 raccoon dogs, as adequate aortic imaging was unavailable in six individuals.

Paired *t*-test results indicated that the right kidney (RK) was significantly shorter than the left kidney (LK; 47.53 ± 3.83 vs. 49.17 ± 3.53 mm, respectively, *p* = 0.001). However, the mean length difference between the two kidneys was 1.62 mm. The mean aortic diameter was 4.74 ± 0.63 mm. The mean length/Ao ratios of the LKs and RKs were 10.53 ± 1.49 and 10.13 ± 1.53, respectively, with a statistically significant difference confirmed by a paired *t*-test (*p* = 0.001). A paired *t*-test revealed no statistically significant difference in RCT between LKs and RKs (4.76 ± 0.49 vs. 4.87 ± 0.66 mm, respectively, *p* > 0.05; Table 1). Therefore, RCT data from both kidneys of 30 raccoon dogs were pooled (*n =* 60) for further analyses.

**Table 1:** RCT and RCT/Ao ratios of the left kidney, right kidney, and both kidneys.

Parameter	Left kidney (*n =* 30)	Right kidney (*n =* 30)	Both kidneys (*n =* 60)
RCT (mm)	4.76 ± 0.49	4.87 ± 0.66	4.81 ± 0.58
RCT/Ao ratio	1.02 ± 0.16	1.04 ± 0.2	1.03 ± 0.18

Data are presented as the mean ± standard deviation. A paired *t*-test revealed no statistically significant difference in RCT or RCT/Ao ratio between the left and right kidneys (*p* > 0.05). RCT, renal cortical thickness; RCT/Ao ratio, RCT-to-aortic diameter ratio.

**Figure 2:**
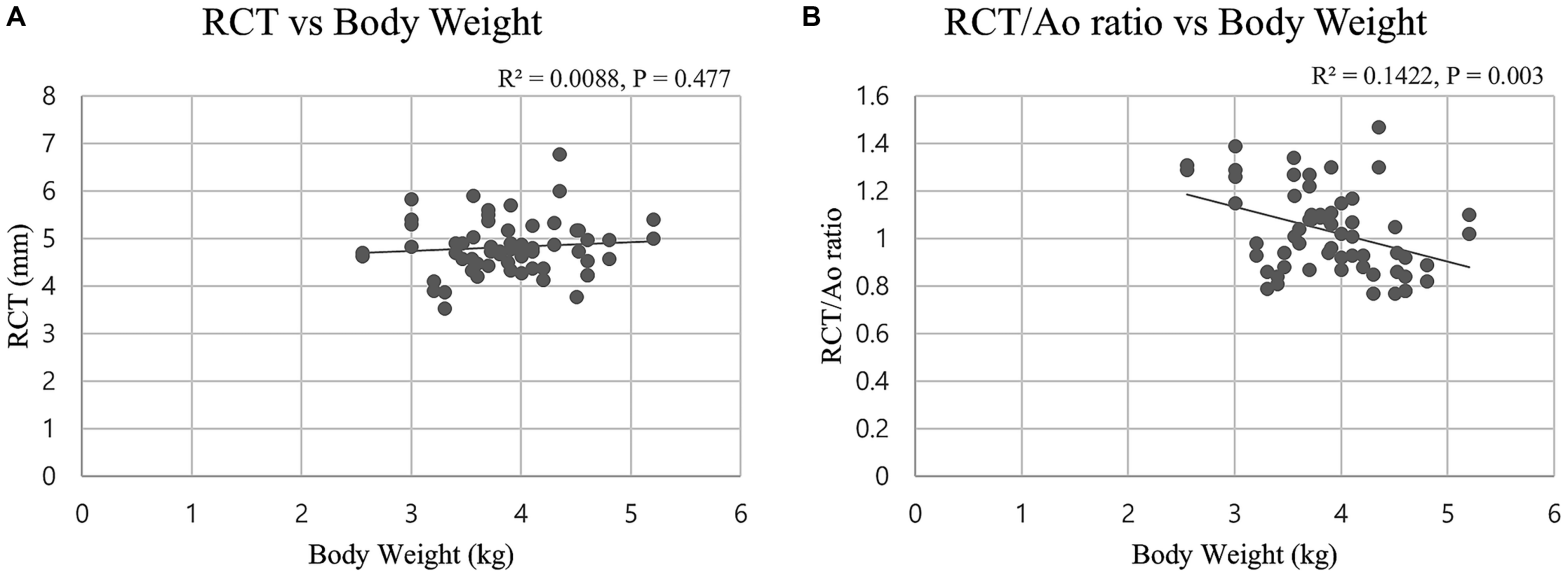
Scatter plots showing the relationships between renal size indices [renal cortical thickness (RCT) and RCT-to-aortic diameter (RCT/Ao) ratio] and body weight in 30 wild Korean raccoon dogs (*n =* 60 kidneys). The lines represent the best-fit linear regressions. **(A)** No significant correlation was found between RCT and body weight (r = 0.094, *p* = 0.477). **(B)** A weak but statistically significant negative correlation was observed between RCT/Ao ratio and body weight (r = −0.377, *p* = 0.003).

Analyses of the effects of sex and BW on renal length and RCT revealed no statistically significant differences (independent *t*-test, *p* > 0.05; Pearson’s correlation, *p* > 0.05; Figure 2). Aortic diameter showed a significant positive correlation with BW (Pearson correlation, *p* < 0.01). In contrast, LK length/Ao, RK length/Ao, and RCT/Ao ratios were significantly negatively correlated with BW (Pearson correlation, *p* < 0.05 for all comparisons; Figure 2), whereas none of these ratios showed statistically significant differences based on sex (independent *t*-test, *p* > 0.05 for all comparisons).

## 4 Discussion

### 4.1 Comparative interpretation of renal parameters

To interpret the renal ultrasonographic parameters measured in Korean raccoon dogs, comparisons were made with previously reported values in dogs and domestic cats (Table 2).

**Table 2:** Comparison of renal ultrasonographic measurements among Korean raccoon dogs, dogs, and domestic cats.

Parameter	Korean raccoon dog (*n =* 36 for length, *n =* 30 for length/Ao ratio, *n =* 60 for RCT, RCT/Ao ratio)	Dog^a,b^ (*n =* 92 for length, *n =* 120 for RCT, *n =* 110 for RCT/Ao ratio, *n =* 92 for length/Ao ratio)	Domestic cat^c^ *n =* 40
Body weight (kg)	4.02 ± 0.73	28.4 ± 16.4^a^ 12.91 ± 9.06^b^	3.98 ± 0.74
LK length (mm)	49.17 ± 3.53	64 ± 15^a^	36.7 ± 4.8
RK length (mm)	47.53 ± 3.83	64 ± 15^a^	38.5 ± 5.0
LK length/Ao ratio	10.53 ± 1.49	7.3 ± 0.9^a,d^	11.69 ± 1.49^d^
RK length/Ao ratio	10.13 ± 1.53	7.3 ± 0.9^a,d^	11.69 ± 1.49^d^
RCT (mm)	4.81 ± 0.58	4.43 ± 1.32^b^	4.7 ± 0.9
RCT/Ao ratio	1.03 ± 0.18	0.70 ± 0.09^b^	–

^a^Mareschal et al. (15). ^b^Lee et al. (7). ^c^Tanvetthayanont et al. (14). ^d^Mean renal length/Ao ratio derived from combined measurements of LKs and RKs. Ao, aortic diameter; LK, left kidney; RCT, renal cortical thickness; RCT/Ao ratio, renal cortical thickness-to-aortic diameter ratio; RK, right kidney.

Korean raccoon dogs exhibited shorter renal lengths but higher length/Ao ratios, RCTs, and RCT/Ao ratios than those observed in dogs. Although dogs, especially large breeds, typically exhibit longer renal lengths due to greater variation in body size and breed diversity, Korean raccoon dogs showed higher values in aorta-indexed renal parameters, such as length/Ao and RCT/Ao ratios. These differences may reflect species-specific anatomical variations in renal and vascular structures within the Canidae family.

In Korean raccoon dogs, the aortic diameter was positively correlated with BW. This trend agrees with previous findings in dogs, where the aortic diameter also increases with BW (13). However, despite this similarity, aorta-based indices such as length/Ao and RCT/Ao ratios may not be directly comparable across species due to differences in overall body conformation and anatomical scaling factors. These differences can influence how renal dimensions relate to aortic size, potentially confounding interspecies interpretations. Therefore, direct interspecies comparisons using aorta-based indices should be approached with caution, and interpretations should consider underlying anatomical and physiological differences between species.

In contrast, Korean raccoon dogs exhibited longer renal lengths but lower length/Ao ratios than those of domestic cats. Although RCT values were similar between Korean raccoon dogs and domestic cats, renal lengths and length/Ao ratios differed, indicating potential anatomical distinctions independent of BW. Nonetheless, caution is warranted, as the length/Ao ratios in Korean raccoon dogs are significantly influenced by BW. Moreover, studies in cats have reported a similar positive correlation between BW and aortic diameter (14), suggesting that aorta-based normalization may not fully account for interspecies differences in body size. These findings support the need to establish species-specific ultrasonographic reference values for Korean raccoon dogs.

### 4.2 Anatomical asymmetry between LKs and RKs

Consistent with a previous study on canine renal measurements (12), the present study also demonstrated a statistically significant difference in renal length between LKs and RKs. The 2.3 mm mean renal length difference was considered clinically negligible in the previous study, given the resolution constraints inherent in ultrasound imaging. In contrast, Mareschal et al. (15) reported no significant difference between LKs and RKs. These discrepancies may be attributable to the use of absolute rather than normalized measurements, as well as methodological differences in imaging protocols or sample population characteristics. Despite the statistically significant difference in renal length, with the RK being shorter than the LK, the mean difference of 1.6 mm observed in the present study was smaller than previously reported values (12) and is unlikely to have clinical significance. Furthermore, the 12-MHz high-frequency ultrasound transducer used in the present study provides superior resolution compared to the lower-frequency transducers employed in earlier studies.

This is particularly relevant because ultrasound lateral resolution improves with frequency, as shown by Ng and Swanevelder (16), indicating that the observed renal length difference likely reflects genuine anatomical variation, rather than being an imaging artifact. In dogs, the RK is often less consistently visualized on ultrasonography because of its cranial position within the rib cage and its location dorsal to the gas-filled intestines, whereas the LK benefits from a more caudal position and the acoustic window provided by the spleen (5).

Although species-specific anatomical studies are lacking, our ultrasonographic and radiographic observations suggest that the RK in Korean raccoon dogs is positioned slightly more cranially than the LK, consistent with the typical arrangement in dogs.

However, the ultrasonographic evaluation of the kidneys in Korean raccoon dogs was not notably hindered by such anatomical limitations, possibly due to species-specific differences in abdominal conformation. Both kidneys shared a similar anatomical orientation, and the RK was not deeply positioned within the rib cage, allowing for consistent and accurate measurements on both sides.

These findings suggest that, given the minimal difference and low likelihood of measurement bias due to asymmetric accessibility, presenting a combined renal length as a reference value is reasonable while acknowledging anatomical asymmetry. Notably, this anatomical observation was based on a limited number of individuals. Therefore, further studies including a larger number of individuals are required to determine whether this ultrasonographic accessibility is consistent across the broader population.

### 4.3 Influence of BW and sex

Although previous studies in dogs have reported statistically significant correlations between renal length and BW, as well as between RCT and BW (7, 12), the present study did not observe such correlations in wild Korean raccoon dogs. This discrepancy may be attributed to inherent differences in body conformation and weight distribution between dogs and wild Korean raccoon dogs. Dogs exhibit significant breed diversity and body size variation (17), whereas Korean raccoon dogs represent a single subspecies with a relatively uniform body conformation and a narrower weight range. Even within a single breed, variations in BW may result from differences in fat accumulation rather than skeletal size.

Such differences in body fat composition can influence renal measurements, such as RCT (7, 18). In the present study, none of the rescued raccoon dogs exhibited clinical signs of obesity, which is consistent with previous reports describing mange-infested individuals as emaciated and dehydrated, often experiencing appetite loss (19). This relatively uniform and low body fat condition in the study population may explain the absence of a significant correlation between BW and renal dimensions.

A similar trend has also been observed in human studies. According to a previous study cited in Lee et al. (7), kidney length was strongly correlated with height in a pediatric population with a wide range of body sizes. Conversely, in adults with a narrower distribution of physiques, no such correlation was found (20). These findings support the observation that significant correlations between BW and renal size may be less evident in physiologically homogeneous populations. However, unlike renal dimensions, aortic diameter was positively correlated with BW in the present study, consistent with findings in cats (14). Although this association has been previously reported, its physiological implications have not been discussed (14). In the present study, this was interpreted with caution because of the potential influence of structural body size and the small sample size.

Renal length and RCT are significantly associated with sex in humans (21, 22). However, no significant correlations were observed between sex and either renal length or RCT in the present study of wild Korean raccoon dogs. Previous studies on dogs noted a small but statistically significant difference in renal length between males and females (12), although this difference was near the resolution limits of ultrasonography, raising questions about its clinical significance.

Unlike humans, where sex-related differences in body morphology are more pronounced, wild Korean raccoon dogs and domestic dogs generally exhibit less sexual dimorphism in their body structures. This reduced sexual dimorphism in body structure likely explains the absence of significant sex-related differences in the renal measurements in this study.

### 4.4 Interpretation of aorta-based indices

The RCT/Ao ratio has been used to evaluate renal size in both dogs and cats (7, 14). Notably, the aortic diameter remains relatively stable even in dehydrated states in both humans and dogs (23–25). Therefore, the RCT/Ao ratio has been proposed as a standardized renal measurement index applicable across various BWs and breeds of dogs (7).

Furthermore, the RCT/Ao ratio changes significantly in response to renal diseases, with a notable decrease in CKD and an increase in AKI (2). These disease-related changes support the use of the RCT/Ao ratio as a potential parameter for the early detection and differentiation of renal disorders.

The present study provides baseline RCT/Ao values for Korean raccoon dogs. Unlike previous findings in dogs, this species exhibited a significant positive correlation between BW and aortic diameter and a negative correlation between BW and the RCT/Ao ratio. These findings suggest that the RCT/Ao ratio may not function as a fully body-size-independent parameter in wild Korean raccoon dogs, highlighting the need for species-specific validation before its clinical application.

### 4.5 Clinical interpretations and limitations

In canine studies, serum creatinine levels have been reported to increase only after more than 75% of renal function is lost (26–28). Accordingly, reliance on serum biochemical markers alone has limitations, and a more comprehensive evaluation incorporating additional clinical assessments is necessary for accurate diagnosis. Previous studies in dogs and cats have consistently demonstrated associations between renal size and renal disease (5).

In addition to renal length, RCT has been investigated as a diagnostic parameter for renal disease, with increasing evidence supporting its clinical relevance. Notably, in dogs with CKD, RCT was significantly lower than in healthy dogs, reflecting cortical atrophy and fibrosis. In contrast, AKI has been associated with an increased RCT due to cortical swelling caused by inflammation. These disease-specific patterns support the potential of RCT as a helpful, noninvasive ultrasonographic parameter that may contribute to distinguishing acute from chronic renal conditions, particularly when serum biochemical changes are equivocal or absent (2).

In this context, the present study aimed to establish baseline ultrasonographic reference values for renal parameters in clinically healthy wild Korean raccoon dogs. Among the parameters assessed, renal length and RCT have been recognized as clinically relevant indicators of renal status in prior research. In our findings, both renal length and RCT were not significantly associated with BW or sex, suggesting their potential reliability as stable ultrasonographic markers in this species.

These results provide a foundation for future research evaluating whether these parameters can effectively differentiate between normal and diseased kidneys in Korean raccoon dogs.

While this study included 36 wild Korean raccoon dogs, the relatively small sample size may limit the generalizability of the findings. Further studies with larger populations are warranted to validate these results.

Although this study included only clinically healthy raccoon dogs without azotemia, additional renal function assessments, such as symmetric dimethylarginine (SDMA) measurement and urinalysis, were not performed. Moreover, a histopathological evaluation was not performed, making it impossible to completely exclude individuals with early or subclinical renal diseases. This limitation should be considered when interpreting the results, and future studies incorporating more comprehensive renal function tests may provide a clearer understanding.

Additionally, due to the nature of wildlife, the exact ages of the animals included in this study could not be determined. Although only weaned adults were enrolled based on physical maturity, the lack of validated age estimation methods for this species limits the ability to assess potential age-related variations in renal parameters.

Another limitation of this study is the lack of a comparison with raccoon dogs known to have renal diseases, such as CKD or AKI. As this study focused on establishing normal reference values in clinically healthy individuals, further research should compare these findings with those from diseased subjects to evaluate the clinical applicability of ultrasonographic renal measurements in diagnosing kidney diseases.

## 5 Conclusion

This study established baseline ultrasonographic reference values for renal length, RCT, and aortic diameter-indexed ratios—length/Ao and RCT/Ao ratios—in clinically healthy wild Korean raccoon dogs. The study also highlighted minimal differences in renal length between LKs and RKs, with anatomical variation unlikely to be clinically significant. No significant correlations between renal measurements and BW or sex were observed, which may reflect the relatively uniform body composition of wild raccoon dogs. These findings suggest that absolute measurements such as renal length and RCT may serve as more stable and clinically useful indices than aorta-based ratios in this species.

## Data Availability

The raw data supporting the conclusions of this article will be made available by the authors, without undue reservation.
